# 2-Methyl-5-nitro­benzene­sulfonamide

**DOI:** 10.1107/S1600536809053069

**Published:** 2009-12-12

**Authors:** Muhammad Zia-ur-Rehman, Islam Ullah Khan, Nargis Naz, Muhammad Nadeem Arshad

**Affiliations:** a Applied Chemistry Research Centre, PCSIR Laboratories Complex, Ferozpure Road, Lahore 54600, Pakistan; b Department of Chemistry, Government College University, Lahore 54000, Pakistan; c Lahore College for Women University, Jail Road, Lahore 54000, Pakistan

## Abstract

In the title compound, C_7_H_8_N_2_O_4_S, the nitro group is twisted by 9.61 (2)° relative to the benzene ring. In the crystal, mol­ecules are linked by N—H⋯O and N—H⋯(O,O) hydrogen bonds between the amino and sulfonyl groups, forming layers parallel to (001).

## Related literature

For the biological activity of sulfonamides, see: Ozbek *et al.* (2007 ▶); Parari *et al.* (2008 ▶); Ratish *et al.* (2009 ▶); Selnam *et al.* (2001 ▶). For related structures, see: Arshad *et al.* (2009 ▶); Gowda *et al.* (2007*a* ▶,*b* ▶,*c* ▶); Khan *et al.* (2009 ▶); Haider *et al.*(2009 ▶). For bond-length data, see: Allen *et al.* (1987 ▶).
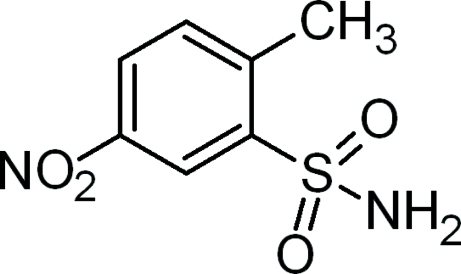


## Experimental

### 

#### Crystal data

C_7_H_8_N_2_O_4_S
*M* *_r_* = 216.21
Orthorhombic, 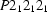

*a* = 4.9872 (4) Å
*b* = 6.2814 (5) Å
*c* = 28.557 (2) Å
*V* = 894.60 (12) Å^3^
*Z* = 4
Mo *K*α radiation
μ = 0.35 mm^−1^
*T* = 296 K
0.43 × 0.17 × 0.11 mm

#### Data collection

Bruker APEXII CCD area-detector diffractometer
Absorption correction: multi-scan (*SADABS*; Sheldrick, 1996 ▶) *T*_min_ = 0.864, *T*_max_ = 0.962
5964 measured reflections
2113 independent reflections
1549 reflections with *I* > 2σ(*I*)
*R*_int_ = 0.036

#### Refinement

*R*[*F* ^2^ > 2σ(*F* ^2^)] = 0.043
*wR*(*F* ^2^) = 0.099
*S* = 0.89
2112 reflections
136 parameters
H atoms treated by a mixture of independent and constrained refinement
Δρ_max_ = 0.23 e Å^−3^
Δρ_min_ = −0.23 e Å^−3^
Absolute structure: Flack (1983 ▶), 766 Friedel pairs
Flack parameter: −0.02 (11)

### 

Data collection: *APEX2* (Bruker, 2007 ▶); cell refinement: *SAINT* (Bruker, 2007 ▶); data reduction: *SAINT*; program(s) used to solve structure: *SHELXS97* (Sheldrick, 2008 ▶); program(s) used to refine structure: *SHELXL97* (Sheldrick, 2008 ▶); molecular graphics: *PLATON* (Spek, 2009 ▶) and *Mercury* (Macrae *et al.*, 2006 ▶); software used to prepare material for publication: *WinGX* (Farrugia, 1999 ▶) and *PLATON*.

## Supplementary Material

Crystal structure: contains datablocks I, global. DOI: 10.1107/S1600536809053069/is2494sup1.cif

Structure factors: contains datablocks I. DOI: 10.1107/S1600536809053069/is2494Isup2.hkl

Additional supplementary materials:  crystallographic information; 3D view; checkCIF report

## Figures and Tables

**Table 1:** Hydrogen-bond geometry (Å, °)

*D*—H⋯*A*	*D*—H	H⋯*A*	*D*⋯*A*	*D*—H⋯*A*
N3—H1*N*⋯O4^i^	0.83 (4)	2.27 (4)	3.055 (4)	158 (3)
N3—H2*N*⋯O4^ii^	0.89 (6)	2.30 (6)	3.107 (4)	150 (4)
N3—H2*N*⋯O3^iii^	0.89 (6)	2.40 (4)	2.893 (4)	115 (3)

Symmetry codes: (i) 
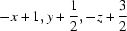
; (ii) 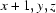; (iii) 
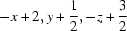
.

**Fig. 1.**
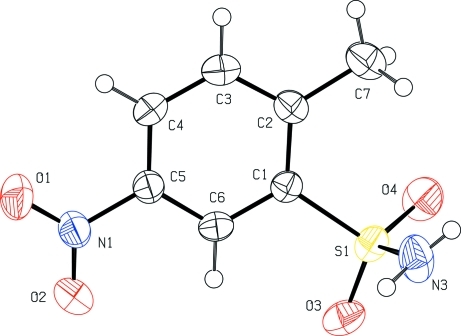
The molecular structure of (I), with displacement ellipsoids at the 50% probability level.

**Fig. 2.**
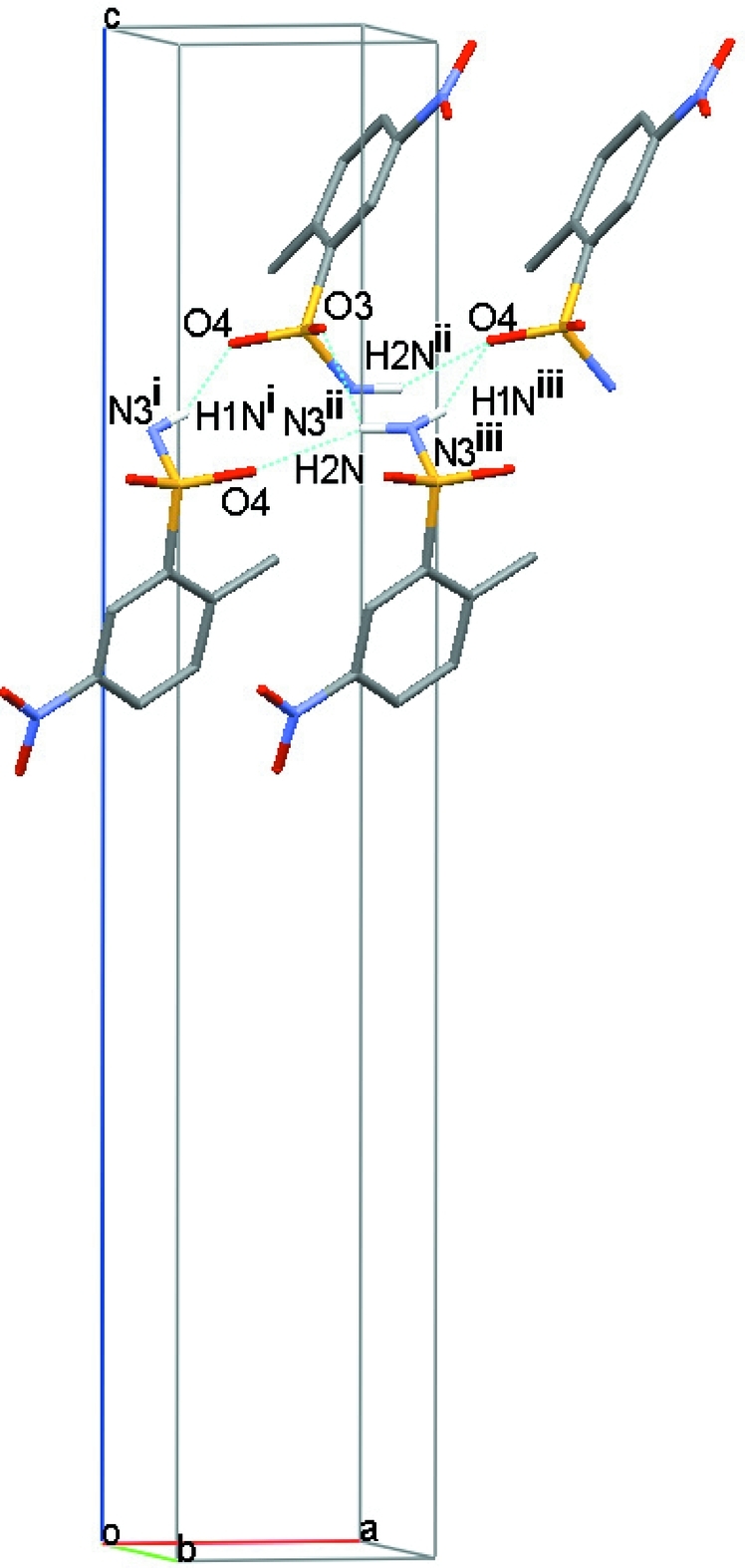
Perspective view of the three-dimensional crystal packing showing intermolecular hydrogen-bonded interactions (dashed lines). [Symmetry codes: (i) -x + 1, y + 1/2, -z + 3/2; (ii) x + 1, y, z and (iii) -x + 2, y + 1/2, -z + 3/2]. H atoms not involved in hydrogen bonding have been omitted for clarity.

## supplementary crystallographic information

### Comment

Sulfonamides are familiar for their anti-HIV (Selnam *et al.*, 2001), anti-inflamatory (Ratish *et al.*, 2009) and anti-microbial (Ozbek *et al.*, 2007; Parari *et al.*, 2008) activities. In continuation of our work regarding the synthesis of various sulfonamides (Arshad *et al.*, 2009; Khan *et al.*, 2009), structure of 2-methyl-5-nitrobenzenesulfonamide (I) has been determined. Bond lengths and bond angles of the title molecule (Fig. 1) are almost similar to those in the related molecules (Gowda *et al.*, 2007*a*,*b*,*c*; Haider *et al.*, 2009) and are within the normal ranges (Allen *et al.*, 1987). Each molecule is linked to its adjacent ones through intermolecular N—H···O hydrogen bonds forming a chain along the *a* axis, while each chain is linked to its neighbouring chain running in opposite direction *via* intermolecular N—H···O═S hydrogen bonds (Table 1 and Fig. 2).

### Experimental

A well ground mixture of 2-methyl-5-nitrobenzenesulfonyl chloride (2.36 g, 10.0 mmol) and ammonium carbonate (10.0 g) was heated in a china dish till the complete removal of typical smell of sulfonyl chloride. Contents were cooled and washed with water followed by crystallization from methanol.

### Refinement

All H atoms were identified in a difference map and then were treated as riding (C—H = 0.93 or 0.97 Å), with *U*_iso_(H) = 1.2*U*_eq_(C). The reflection '0 0 2' affected by beamstop was removed during refinement.

### Crystal data

**Table:**

C_7_H_8_N_2_O_4_S	*F*(000) = 448
*M**_r_* = 216.21	*D*_x_ = 1.605 Mg m^−^^3^
Orthorhombic, *P*2_1_2_1_2_1_	Mo *K*α radiation, λ = 0.71073 Å
Hall symbol: P 2ac 2ab	Cell parameters from 1325 reflections
*a* = 4.9872 (4) Å	θ = 2.9–25.4°
*b* = 6.2814 (5) Å	µ = 0.35 mm^−^^1^
*c* = 28.557 (2) Å	*T* = 296 K
*V* = 894.60 (12) Å^3^	Needles, colourless
*Z* = 4	0.43 × 0.17 × 0.11 mm

### Data collection

**Table:**

Bruker APEXII CCD area-detector diffractometer	2113 independent reflections
Radiation source: fine-focus sealed tube	1549 reflections with *I* > 2σ(*I*)
graphite	*R*_int_ = 0.036
φ and ω scans	θ_max_ = 28.3°, θ_min_ = 1.4°
Absorption correction: multi-scan (*SADABS*; Sheldrick, 1996)	*h* = −3→6
*T*_min_ = 0.864, *T*_max_ = 0.962	*k* = −8→8
5964 measured reflections	*l* = −38→38

### Refinement

**Table:**

Refinement on *F*^2^	Secondary atom site location: difference Fourier map
Least-squares matrix: full	Hydrogen site location: inferred from neighbouring sites
*R*[*F*^2^ > 2σ(*F*^2^)] = 0.043	H atoms treated by a mixture of independent and constrained refinement
*wR*(*F*^2^) = 0.099	*w* = 1/[σ^2^(*F*_o_^2^) + (0.0508*P*)^2^ + 0.1986*P*] where *P* = (*F*_o_^2^ + 2*F*_c_^2^)/3
*S* = 0.89	(Δ/σ)_max_ < 0.001
2112 reflections	Δρ_max_ = 0.23 e Å^−^^3^
136 parameters	Δρ_min_ = −0.23 e Å^−^^3^
0 restraints	Absolute structure: Flack (1983), 766 Friedel pairs
Primary atom site location: structure-invariant direct methods	Flack parameter: −0.02 (11)

### Special details

**Table:**

Geometry. All s.u.'s (except the s.u. in the dihedral angle between two l.s. planes) are estimated using the full covariance matrix. The cell s.u.'s are taken into account individually in the estimation of s.u.'s in distances, angles and torsion angles; correlations between s.u.'s in cell parameters are only used when they are defined by crystal symmetry. An approximate (isotropic) treatment of cell s.u.'s is used for estimating s.u.'s involving l.s. planes.
Refinement. Refinement of *F*^2^ against ALL reflections. The weighted *R*-factor *wR* and goodness of fit *S* are based on *F*^2^, conventional *R*-factors *R* are based on *F*, with *F* set to zero for negative *F*^2^. The threshold expression of *F*^2^ > 2σ(*F*^2^) is used only for calculating *R*-factors(gt) *etc*. and is not relevant to the choice of reflections for refinement. *R*-factors based on *F*^2^ are statistically about twice as large as those based on *F*, and *R*- factors based on ALL data will be even larger.

### Fractional atomic coordinates and isotropic or equivalent isotropic displacement parameters (Å^2^)

**Table:**

	*x*	*y*	*z*	*U*_iso_*/*U*_eq_	
C1	0.7436 (6)	0.4718 (4)	0.85981 (8)	0.0294 (6)	
C2	0.6274 (6)	0.6621 (4)	0.87573 (9)	0.0336 (6)	
C3	0.7125 (7)	0.7374 (4)	0.91916 (10)	0.0433 (8)	
H3	0.6392	0.8629	0.9308	0.052*	
C4	0.9018 (6)	0.6316 (5)	0.94542 (9)	0.0425 (7)	
H4	0.9568	0.6857	0.9742	0.051*	
C5	1.0083 (7)	0.4455 (4)	0.92867 (9)	0.0342 (6)	
C6	0.9298 (6)	0.3616 (4)	0.88605 (8)	0.0330 (6)	
H6	1.0009	0.2338	0.8753	0.040*	
C7	0.4234 (7)	0.7867 (5)	0.84858 (11)	0.0473 (9)	
H7A	0.3861	0.9178	0.8646	0.071*	
H7B	0.2615	0.7051	0.8459	0.071*	
H7C	0.4922	0.8171	0.8179	0.071*	
N1	1.2116 (5)	0.3335 (4)	0.95582 (8)	0.0431 (6)	
O4	0.3903 (4)	0.3752 (4)	0.79524 (7)	0.0525 (6)	
O1	1.3007 (5)	0.4194 (3)	0.99090 (7)	0.0568 (6)	
O2	1.2851 (5)	0.1593 (4)	0.94205 (8)	0.0677 (7)	
O3	0.7986 (4)	0.1599 (3)	0.80208 (7)	0.0465 (5)	
N3	0.8152 (8)	0.5117 (5)	0.76612 (9)	0.0532 (8)	
S1	0.67301 (15)	0.36352 (11)	0.80358 (2)	0.03595 (19)	
H1N	0.720 (8)	0.605 (5)	0.7540 (12)	0.062 (12)*	
H2N	0.994 (12)	0.509 (7)	0.7660 (14)	0.091 (16)*	

### Atomic displacement parameters (Å^2^)

**Table:**

	*U*^11^	*U*^22^	*U*^33^	*U*^12^	*U*^13^	*U*^23^
C1	0.0257 (18)	0.0334 (12)	0.0292 (11)	−0.0031 (12)	0.0030 (11)	0.0005 (10)
C2	0.0294 (18)	0.0338 (13)	0.0374 (12)	−0.0008 (13)	0.0030 (11)	0.0044 (11)
C3	0.046 (2)	0.0403 (14)	0.0437 (15)	0.0085 (15)	0.0036 (15)	−0.0086 (12)
C4	0.047 (2)	0.0469 (15)	0.0334 (12)	0.0080 (17)	−0.0022 (13)	−0.0102 (13)
C5	0.0295 (18)	0.0429 (14)	0.0301 (12)	0.0029 (13)	0.0029 (12)	0.0031 (11)
C6	0.0313 (17)	0.0345 (12)	0.0333 (12)	0.0013 (14)	0.0065 (11)	−0.0029 (12)
C7	0.045 (2)	0.0428 (16)	0.0541 (17)	0.0074 (15)	0.0010 (16)	0.0078 (13)
N1	0.0377 (16)	0.0553 (14)	0.0362 (11)	0.0095 (15)	−0.0022 (11)	0.0052 (11)
O4	0.0291 (12)	0.0768 (14)	0.0515 (12)	−0.0103 (12)	−0.0018 (9)	−0.0106 (11)
O1	0.0521 (16)	0.0746 (15)	0.0436 (11)	0.0029 (13)	−0.0166 (11)	−0.0034 (10)
O2	0.0711 (19)	0.0721 (14)	0.0598 (14)	0.0387 (16)	−0.0127 (13)	−0.0080 (12)
O3	0.0495 (14)	0.0433 (10)	0.0467 (10)	−0.0050 (11)	0.0040 (10)	−0.0132 (9)
N3	0.039 (2)	0.079 (2)	0.0415 (13)	−0.0008 (19)	0.0032 (15)	0.0203 (14)
S1	0.0296 (4)	0.0473 (4)	0.0310 (3)	−0.0068 (4)	0.0021 (3)	−0.0038 (3)

### Geometric parameters (Å, °)

**Table:**

C1—C6	1.379 (4)	C6—H6	0.9300
C1—C2	1.404 (4)	C7—H7A	0.9600
C1—S1	1.779 (3)	C7—H7B	0.9600
C2—C3	1.393 (4)	C7—H7C	0.9600
C2—C7	1.499 (4)	N1—O1	1.221 (3)
C3—C4	1.377 (4)	N1—O2	1.219 (3)
C3—H3	0.9300	O4—S1	1.432 (2)
C4—C5	1.370 (4)	O3—S1	1.425 (2)
C4—H4	0.9300	N3—S1	1.586 (3)
C5—C6	1.383 (3)	N3—H1N	0.83 (4)
C5—N1	1.457 (4)	N3—H2N	0.89 (6)
			
C6—C1—C2	122.0 (2)	C2—C7—H7A	109.5
C6—C1—S1	115.57 (19)	C2—C7—H7B	109.5
C2—C1—S1	122.4 (2)	H7A—C7—H7B	109.5
C3—C2—C1	116.8 (2)	C2—C7—H7C	109.5
C3—C2—C7	119.3 (3)	H7A—C7—H7C	109.5
C1—C2—C7	123.9 (2)	H7B—C7—H7C	109.5
C4—C3—C2	122.0 (3)	O1—N1—O2	123.5 (3)
C4—C3—H3	119.0	O1—N1—C5	118.5 (2)
C2—C3—H3	119.0	O2—N1—C5	118.1 (2)
C5—C4—C3	119.2 (3)	S1—N3—H1N	116 (3)
C5—C4—H4	120.4	S1—N3—H2N	116 (3)
C3—C4—H4	120.4	H1N—N3—H2N	126 (4)
C4—C5—C6	121.5 (3)	O3—S1—O4	118.28 (15)
C4—C5—N1	119.7 (2)	O3—S1—N3	108.08 (18)
C6—C5—N1	118.8 (2)	O4—S1—N3	107.33 (18)
C1—C6—C5	118.5 (2)	O3—S1—C1	106.47 (12)
C1—C6—H6	120.7	O4—S1—C1	108.99 (13)
C5—C6—H6	120.7	N3—S1—C1	107.21 (15)
			
C6—C1—C2—C3	1.2 (4)	N1—C5—C6—C1	−177.9 (2)
S1—C1—C2—C3	−175.9 (2)	C4—C5—N1—O1	−6.9 (4)
C6—C1—C2—C7	−179.7 (3)	C6—C5—N1—O1	172.3 (3)
S1—C1—C2—C7	3.3 (4)	C4—C5—N1—O2	173.7 (3)
C1—C2—C3—C4	0.1 (4)	C6—C5—N1—O2	−7.1 (4)
C7—C2—C3—C4	−179.1 (3)	C6—C1—S1—O3	9.8 (2)
C2—C3—C4—C5	−0.6 (5)	C2—C1—S1—O3	−173.0 (2)
C3—C4—C5—C6	0.0 (4)	C6—C1—S1—O4	138.5 (2)
C3—C4—C5—N1	179.2 (3)	C2—C1—S1—O4	−44.3 (3)
C2—C1—C6—C5	−1.8 (4)	C6—C1—S1—N3	−105.7 (2)
S1—C1—C6—C5	175.4 (2)	C2—C1—S1—N3	71.6 (3)
C4—C5—C6—C1	1.3 (4)		

### Hydrogen-bond geometry (Å, °)

**Table:**

*D*—H···*A*	*D*—H	H···*A*	*D*···*A*	*D*—H···*A*
N3—H1N···O4^i^	0.83 (4)	2.27 (4)	3.055 (4)	158 (3)
N3—H2N···O4^ii^	0.89 (6)	2.30 (6)	3.107 (4)	150 (4)
N3—H2N···O3^iii^	0.89 (6)	2.40 (4)	2.893 (4)	115 (3)

Symmetry codes: (i) −*x*+1, *y*+1/2, −*z*+3/2; (ii) *x*+1, *y*, *z*; (iii) −*x*+2, *y*+1/2, −*z*+3/2.
